# Impact of down-stream processing on functional properties of yeasts and the implications on gut health of Atlantic salmon (*Salmo salar*)

**DOI:** 10.1038/s41598-021-83764-2

**Published:** 2021-02-24

**Authors:** Jeleel Opeyemi Agboola, Marion Schiavone, Margareth Øverland, Byron Morales-Lange, Leidy Lagos, Magnus Øverlie Arntzen, David Lapeña, Vincent G. H. Eijsink, Svein Jarle Horn, Liv Torunn Mydland, Jean Marie François, Luis Mercado, Jon Øvrum Hansen

**Affiliations:** 1 Department of Animal and Aquacultural Sciences, Norwegian University of Life Sciences, P.O. Box 5003, 1432 Ås, Norway; 2 Lallemand SAS, 19 rue des Briquetiers, BP59, 31702 Blagnac, France; 3 Faculty of Chemistry, Biotechnology and Food Science, Norwegian University of Life Sciences, P.O. Box 5003, 1432 Ås, Norway; 4 TBI, Université de Toulouse, CNRS, INRAE, INSA, Toulouse, France; 5 Grupo de Marcadores Inmunológicos en Organismos Acuáticos, Pontificia Universidad Católica de Valparaíso, Avenida Universidad 330, Valparaíso, Chile; 6 LAAS-CNRS, Université de Toulouse, CNRS, Toulouse, France

**Keywords:** Tumour-necrosis factors, Atomic force microscopy, Immunochemistry, Ulcerative colitis, ELISA, Immunohistochemistry

## Abstract

Yeasts are becoming popular as novel ingredients in fish feeds because of their potential to support better growth and concomitantly ensure good fish health. Here, three species of yeasts (*Cyberlindnera jadinii*, *Blastobotrys adeninivorans* and *Wickerhamomyces anomalus*), grown on wood sugars and hydrolysates of chicken were subjected to two down-stream processes, either direct heat-inactivation or autolysis, and the feed potential of the resulting yeast preparations was assessed through a feeding trial with Atlantic salmon fry. Histological examination of distal intestine based on widening of lamina propria, showed that autolyzed *W. anomalus* was effective in alleviating mild intestinal enteritis, while only limited effects were observed for other yeasts. Our results showed that the functionality of yeast in counteracting intestinal enteritis in Atlantic salmon was dependent on both the type of yeast and the down-stream processing method, and demonstrated that *C. jadinii* and *W. anomalus* have promising effects on gut health of Atlantic salmon.

## Introduction

Future growth of salmon farming is highly dependent on sustainable feed ingredients that meet the nutritional needs and improve overall health status of fish, at low environmental cost. The growth of the salmon sector imposes demands on feed resources like wild fish stocks, which are under pressure^1,2^. This has led to a change in the salmon feed composition, from being mainly based on marine ingredients towards the use of more plant-based ingredients^3^. Studies have shown that high dietary inclusion of plant ingredients such as soybean meal (SBM)^4–10^, pea protein concentrate^11^, faba bean^12,13^ and corn gluten meal^14^, is associated with a condition widely known as SBM induced enteritis (SBMIE) in fish, including Atlantic salmon, rainbow trout and sea bass.

Microbial ingredients such as yeasts^15,16^, bacterial meal^17–19^ and microalgae^15^ have been shown to counteract SBMIE in Atlantic salmon. However, question remains on whether this effect is primarily due to the intrinsic properties of microbial biomass itself, the type of processing or the combination of both. Øverland and Skrede^20^ suggested that down-stream processing of yeast after harvesting is imperative to preserve valuable nutrients and bioactive components, and to improve nutrient digestibility in fish. Previously, chemical, enzymatic, physical, and mechanical treatments have been used to enhance the nutritional and functional values of yeasts for various applications^21–24^. Different down-stream processing strategies have shown varying impacts on the integrity and nutritional values of yeast^21,23^. While down-stream processing may increase accessibility to contents of the yeasts cells, methods such as cell crushing using a microfluidizer may be excessively harsh, leading to alteration in bioavailability of the bioactive components^25,26^. Having this in mind and considering cost-effectiveness in terms of energy savings, scalability and commercialization, autolysis was selected as the down-stream processing method in the present study.

Autolysis is a slow process during which cell membrane permeability increases and endogenous lytic enzymes such as proteases, β-glucanases and chitinases are activated within the yeast cells^27,28^, leading to lysis of the intracellular components of the cell. Autolysis can be induced at low pH or high temperature^21,23,29–32^. Hernawan and Fleet^29^ reported that the ultrastructure and content of yeast cell wall polysaccharides could be modified through autolysis. In addition, using atomic force microscopy (AFM), Schiavone, et al.^32^ have shown that autolysis can be used to enhance the adhesive property of mannoprotein present on yeast cell wall. However, to our knowledge, no study has reported the relationship between changes induced by autolysis and the effectiveness of yeast in modulating gut health in Atlantic salmon fry.

The current study was designed to investigate whether the ability of yeast to counteract enteritis is linked to either the type of yeast, with its associated cell wall properties, or to the down-stream processing used during yeast production, or a combination of both factors. To address this question, we used three different non-*Saccharomyces* yeasts, namely *Cyberlindnera jadinii* (anamorph name *Candida utilis*)*, Blastobotrys adeninivorans* (synonym *Arxula adeninivorans*) and *Wickerhamomyces anomalus* produced at laboratory scale. The yeasts were selected based on their ability to utilize hydrolyzates of wood and meat co-products, their high growth rate and high protein content as well as their low production of side products such as alcohol^22,33^. Yeasts were subjected to direct inactivation or autolysis and their functionality as feed ingredient was tested using 5% inclusion levels in diets for Atlantic salmon fry. The impact of the down-stream processing on the yeast cells and the impact of the yeast cells on salmon performance were assessed using a variety of methods.

## Results

### Production of yeast

The three types of yeast were produced by fermentation at 20 or 200 L scale using a growth medium based on wood-derived sugars and a hydrolysate of by-products from chicken^22^. The yeast cells were harvested and washed before either spray-drying directly or autolysis followed by spray-drying. Table 1 shows compositional data for the various yeast preparations. The contents (% of dry mass) of the cell wall components β-glucan, mannan, and chitin ranged between 9.6% and 20.4%, 8.7% and 17.8%, and 1.1% and 2.7%, respectively. The total glucan content of *C. jadinii* was 40–80% higher compared to *B. adeninivorans* and *W. anomalus*. On the other hand, the mannan and chitin contents of *W. anomalus* were 30–40% and 17–60% higher compared to *C. jadinii* and *B. adeninivorans*, respectively. Autolysis reduced the glucan content by 20%, 13% and 18% for *C. jadinii, W. anomalus* and *B. adeninivorans,* respectively. There was no reduction in mannan content after autolysis for *C. jadinii,* whereas the mannan contents of the other yeasts were reduced by 5 to 15%. The chitin content was lower in autolyzed yeasts compared to inactivated yeasts, except for *B. adeninivorans*.

**Table 1:** Composition of yeast cells with and without autolysis treatment, after the drying process^a^.

Yeast species^b^	*Cyberlindnera jadinii*		*Blastobotrys adeninivorans*		*Wickerhamomyces anomalus*	
	Inactivated	Autolyzed	Inactivated	Autolyzed	Inactivated	Autolyzed
Dry matter^c^	94.0 ± 0.01	92.4 ± 0.10	95.3 ± 0.05	94.3 ± 0.11	94.9 ± 0.00	93.6 ± 0.20
**Cell wall polysaccharides (% dry mass)**^**c**^						
α-glucan	2.0 ± 0.04	1.3 ± 0.06	0.7 ± 0.48	0.3 ± 0.01	0.3 ± 0.03	0.3 ± 0.01
β-glucan	20.4 ± 1.71	16.0 ± 1.30	12.3 ± 1.61	10.1 ± 0.92	11.1 ± 0.88	9.6 ± 0.71
Mannan	10.9 ± 0.58	11.5 ± 0.77	10.2 ± 0.80	8.7 ± 0.57	17.8 ± 1.36	16.7 ± 0.91
Chitin	1.1 ± 0.14	1.9 ± 0.08	2.2 ± 0.08	2.1 ± 0.29	2.0 ± 0.24	2.7 ± 0.33
**Other components (% dry mass)**^**c**^						
Crude protein	45.6 ± 0.05	47.6 ± 0.09	38.9 ± 0.07	37.4 ± 0.09	52.8 ± 0.05	52.8 ± 0.21
Crude lipids	6.0 ± 0.00	6.2 ± 0.00	8.6 ± 0.00	8.5 ± 0.00	8.8 ± 0.00	9.1 ± 0.02
Ash	7.8 ± 0.04	8.1 ± 0.01	6.1 ± 0.02	6.3 ± 0.00	3.3 ± 0.00	3.2 ± 0.04
Sum of analyzed components^c^	93.8	92.7	78.9	73.4	96.2	94.4

^a^Cell wall thickness (nm): Inactivated *Cyberlindnera jadinii* = 95.9, autolyzed *C. jadinii* = 80.9, inactivated *Blastobotrys adeninivorans* = 103.6, autolyzed *B. adeninivorans* = 62.2, inactivated *Wickerhamomyces anomalus* = 161.4 and autolyzed *W. anomalus* = 116.5.

^b^Composition of ref-*Cyberlindnera jadinii* (% dry weight): α-glucan = 0.9, β-glucan = 15.0, mannans = 9.4, chitin = 1.9, dry matter = 93.2, crude protein = 58.1, crude lipids = 7.0 and ash = 5.4. This is the same yeast used in Grammes et al.^15^.

^c^Amounts of α-glucans, β-glucans and mannans of the cell wall are mean value ± SD from triplicate analyses; whereas chitin, dry matter, crude protein, crude lipids and ash were from duplicate analyses.

^d^Sum of analyzed components is equal to sum of the cell wall polysaccharides and other components in the yeast ingredients.

The contents of crude protein, crude lipids and ash were mostly unaffected by autolysis (Table 1). *W. anomalus* had the highest crude protein (52–53%) and crude lipids content (8–9%) compared to *C. jadinii* (45–48% for crude protein and 6–6.2% for crude lipids) and *B*. *adeninivorans* (37–39% for crude protein and 8.5–8.6% for crude lipids). The ash contents ranged from 3–8%. The sum of detected compounds showed values close to 100% for *C. jadinii* (93.2%) and *W. anomalus* (95.3%), whereas this value was clearly lower for *B. adeninivorans* (76.1%), suggesting that *B. adeninivorans* contained components that were underestimated and/or undetected by our analysis (Table 1).

### Fish growth performance

To assess the impact of the yeasts on SBMIE in Atlantic salmon fry, a feeding trial was conducted where fish were raised from an average initial weight of 5 to 25 g during the experimental period (Supplementary Fig. S3a). During this period, no mortality or abnormal behaviour were observed. There were no significant differences in feed intake, biomass gain and SGR (*P* > *0.05*) between the various dietary treatments (Supplementary Fig. S3b, d). The FM fed fish had lower FCR compared to the other dietary treatments (Supplementary Fig. S3c).

### Morphology and histopathological changes in fish

**Figure 1:**
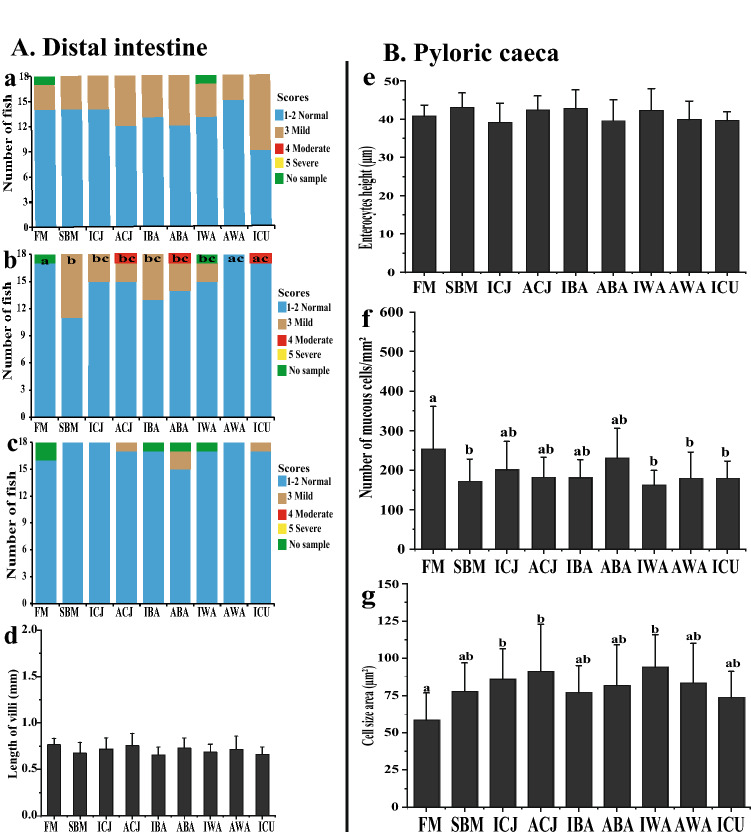
Morphological and histopathological changes in the distal intestine (**A**) and pyloric caeca (**B**) of Atlantic salmon fry fed FM-based diet or SBM-based diets with yeasts. The histological scores were obtained through a semi-quantitative scoring system measuring changes in three morphological parameters: (**a**) loss of supranuclear vacuoles in absorptive enterocytes; (**b**) widening of the lamina propria in mucosal folds; (**c**) increase of connective tissue between base of folds and stratum compactum; and measurement of villi length (**d**). Each parameter (**a**–**c**) was given a score of “**1**–**2**” representing normal morphology; “**3**–**4**” mild and moderate enteritis; whereas “**5**” denotes severe enteritis. For changes in pyloric caeca, the enterocyte height (**e**), the number of mucous cells/mm^2^ mucosal area (**f**) and average mucous cell size (**g**) in the mucosal area are presented. Groups with different letters (**a**–**c**) above the bar charts are significantly different (*P* < *0.05*). The **green bar** represents the number of missing fish samples. The diets are: **FM**-fishmeal-based; **SBM**-Soybean meal-based; 7 other diets containing 40% SBM and 5% of inactivated *Cyberlindnera jadinii* (**ICJ**), autolyzed *C. jadinii* (**ACJ**), inactivated *Blastobotrys adeninivorans* (**IBA**), autolyzed *B. adeninivorans* (**ABA**), inactivated *Wickerhamomyces anomalus* (**IWA**), autolyzed *W. anomalus* (**AWA**) and ref-*C. jadinii* (**ICU**).

Fish fed with the FM diet showed normal distal intestine morphology, whereas fish fed the SBM diet developed mild signs of SBMIE (Fig. 1b and Supplementary Fig. S2a, b). Considering widening of the lamina propria, fish fed the AWA or ICU diet showed only mild signs of SBMIE that were not statistically different (*P* > *0.05*) from the FM treatment (Fig. 1b). Thus, based on this parameter, the AWA and ICU diets led to suppression of SBMIE in the SBM control diet. Fish fed either the ACJ (*P* = 0.072) or IWA (*P* = 0.067) diets showed mild signs of SBMIE in the distal intestine and showed a tendency to be statistically distinguishable from the SBM group (Fig. 1b). Fish fed either ICJ, IBA or ABA were not statistically different from the SBM control. When considering changes in supranuclear vacuoles (Fig. 1a) and connective tissue (Fig. 1c) in absorptive enterocytes, there were no differences between the diets. Morphometric measurements of villi length showed there was no significant difference among the diets (Fig. 1d). Similarly, there was no significant variation in the morphological measurement of the enterocyte height of the pyloric caeca among the diets (Fig. 1e). Also, the number and size of mucous cells in the mucosal area of pyloric caeca were significantly different between the diets (Fig. 1f, g).

### Changes in immune response parameters

**Figure 2:**
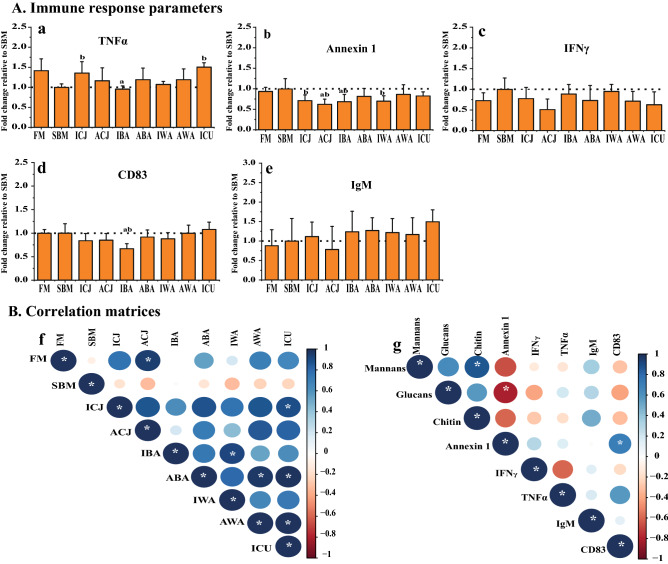
Immune responses (**A**) of Atlantic salmon fry fed soybean meal-based diets with yeasts. Protein expression values for distal intestine (**a**–**e**) were obtained by indirect ELISA and are expressed as fold-change relative to the value obtained for the SBM group. Correlation matrices between the immune markers, and calculated mannan, glucan and chitin intake are shown in panel (**B**). Graph (**f**) shows the correlation between all the experimental diets using five immunological markers (**TNFα**, **Annexin 1**, **IFNγ, CD83 and IgM**). Graph (**g**) shows the correlation between the calculated average daily intake of glucan, mannan and chitin, and the previously mentioned immune markers. The average daily intake of glucans, mannans and chitin were calculated from average dry matter daily feed intake and the composition of the respective cell wall components in each yeast (Table 1). For the correlation matrices, **positive** correlations are displayed in **blue** and **negative** correlations in **red** color; both the **color intensity** and the **size of the circle** are proportional to the correlation coefficients. The diets are: **FM**-fishmeal-based; **SBM**-Soybean meal-based; 7 other diets containing 40% SBM and 5% of inactivated *Cyberlindnera jadinii* (**ICJ**), autolyzed *C. jadinii* (**ACJ**), inactivated *Blastobotrys adeninivorans* (**IBA**), autolyzed *B. adeninivorans* (**ABA**), inactivated *Wickerhamomyces anomalus* (**IWA**), autolyzed *W. anomalus* (**AWA**) and ref-*C. jadinii* (**ICU**). The letters **a** and **b** directly above the bar charts (**a**–**e**) denote treatment(s) with a statistical difference (*P* < *0.05*) compared to the fishmeal and soybean meal control groups, respectively. Correlations (**f**, **g**) with significant values at *P* < *0.05* are shown with *.

Measurements of protein expression of five different markers indicative of immunological response in the distal intestine showed significant differences between the FM and SBM control diets for TNFα and Annexin 1 (Fig. 2a, b). There was a significant increase (*P* < *0.05*) in the level of TNFα in fish fed the ICJ and ICU diets compared to the SBM control (Fig. 2a). In addition, fish fed the yeast diets ACJ and IBA showed significantly reduced levels of Annexin 1, relative to the SBM control (Fig. 2b). No significant differences were observed in the level of CD83 for all the experimental diets, except for the IBA diet, which gave a significantly lower CD83 level compared to both the FM and SBM controls (Fig. 2d). No significant differences were detected in the levels of IFNγ and IgM (Fig. 2c, e).

Relationships among all diets based on the five immune markers, showed a positive and significant correlation (*P* < *0.05*) between the FM control diet and the ACJ diet (Fig. 2f). Also, several of the experimental diets (ICJ/ICU, IBA/IWA, ABA/AWA, ABA/ICU and AWA/ICU) showed significant positive correlations (*P* < 0.05) (Fig. 2f). Furthermore, we examined possible correlations between cell wall components of yeasts consumed by the fish and the measured immune parameters. The daily intake of glucans, mannans and chitin was calculated from average daily feed intake (dry matter) and the presence of the respective cell wall components in each yeast (Table 1). Based on these calculations, fish fed ICJ/ACJ consumed the highest amounts of glucans (3–4 mg per day), whereas fish fed IWA/AWA had the highest intake of mannans (3–3.2 mg per day) (Supplementary Table S3). The results of the correlation analysis showed a negative and significant relationship between Annexin 1 expression in the distal intestine and glucan intake (Fig. 2g).

### Yeast ultrastructure and cell wall composition

**Figure 3:**
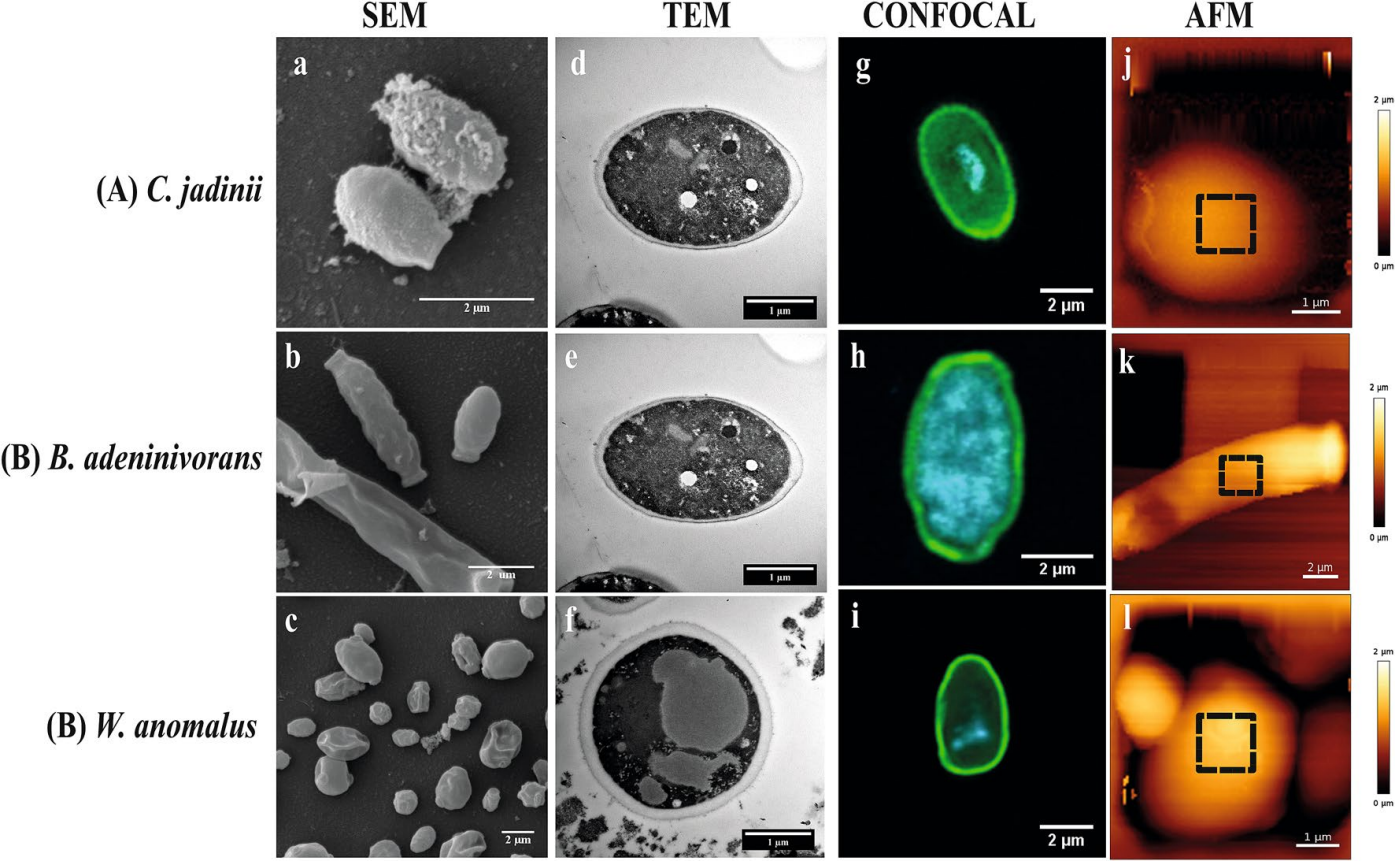
Cell surface architecture of three inactivated yeast species grown on sugars from lignocellulosic biomass. The pictures show Scanning Electron Microscopy (**SEM**; **a**–**c**), Transmission Electron Microscopy (**TEM**; **d**–**f**), Confocal microscopy (stained with concanavalin A-FITC for mannan) (**g**–**i**) and Atomic Force Microscopy (**AFM**; height) (**j**–**l**) micrographs of *Cyberlindnera jadinii* (**panel A**), *Blastobotrys adeninivorans* (**panel B**) and *Wickerhamomyces anomalus* (**panel C**). The SEM and TEM micrographs were taken on yeast creams (before drying), whereas the confocal and AFM micrographs were taken on dried yeast samples, as described in the ‘Material and Methods’. The dotted squares on the AFM height micrographs represent the spots where mapping was done for determination of the Young modulus and measurement of adhesion events, as described in ‘Material and Methods’.

**Figure 4:**
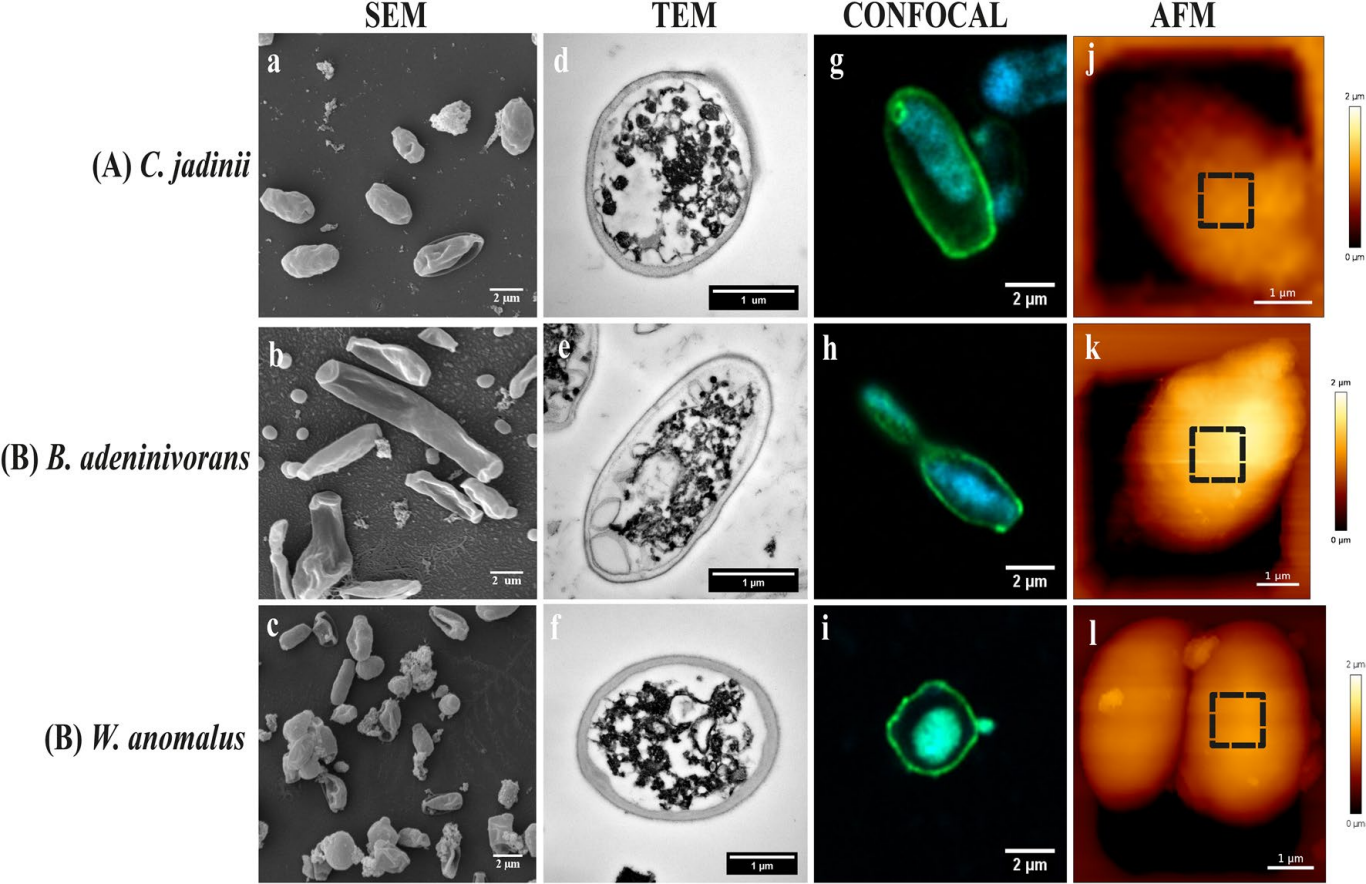
Cell surface architecture of three autolyzed yeast species (50 °C for 16 h) grown on sugars from lignocellulosic biomass. The pictures show Scanning Electron Microscopy (**SEM**; **a**–**c**), Transmission Electron Microscopy (**TEM**; **d**–**f**), Confocal microscopy (stained with concanavalin A-FITC for mannan) (**g**–**i**) and Atomic Force Microscopy (**AFM**; height) (**j**–**l**) micrographs of *Cyberlindnera jadinii* (**panel A**), *Blastobotrys adeninivorans* (**panel B**) and *Wickerhamomyces anomalus* (**panel C**). The SEM and TEM micrographs were taken on yeast creams (before drying), whereas the confocal and AFM micrographs were taken on dried yeast samples, as described in the ‘Material and Methods’. The dotted squares on the AFM height micrographs represent the spots where mapping was done for determination of the Young modulus and measurement of adhesion events, as described in ‘Material and Methods’.

SEM micrographs (Figs. 3 and 4) showed that *C. jadinii* and *W. anomalus* have ovoid-like shape, whereas *B. adeninivorans* has a rod-like shape. The inactivated yeasts (Fig. 3a–c) appeared to have smooth surfaces with no wrinkles, whereas the autolyzed yeasts (Fig. 4a–c) appeared shrivelled and partly broken, seemingly releasing their intracellular contents. These observations were further confirmed by the TEM micrographs, which showed that the intracellular compartment in inactivated cells (Fig. 3d–f) is compact, with visible organelles, whereas autolyzed cells showed a destroyed intracellular structure (Fig. 4d–f). Measurements of cell wall thickness showed that, among the three inactivated yeasts, *W. anomalus* had the thickest cell wall (ca. 160 nm), followed by *B. adeninivorans* (ca. 104 nm) and *C. jadinii* (ca. 96 nm), which both had considerably thinner cell walls (Table 1). Autolysis reduced the cell wall thickness of all the three yeasts, but the extent of this reduction varied. *B. adeninivorans* was mostly affected by autolysis, showing a reduction of nearly 40% in cell wall thickness. For *W. anomalus* and *C. jadinii* the reductions were 28% and 16%, respectively (Table 1). Confocal and AFM imaging showed that yeasts were able to retain their shape and structure after the drying process. After the spray-drying process (Fig. 3g–l), yeasts regained their shape like yeast creams i.e. before drying (Fig. 3a–f) and appeared smooth with thicker and intact intracellular layers. In comparison, autolyzed dry yeasts appear roughened and possess thinner and hollow intracellular layers (Fig. 4g–l).

### Yeast surface properties determined by AFM

**Figure 5:**
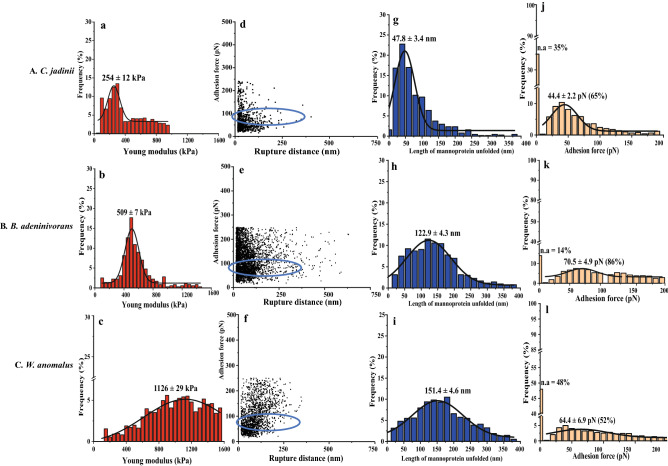
Probing cell wall architecture of three inactivated yeast species (**A**–**C**) with Atomic Force Microscopy, using naked tips (**a**–**c**) or tips functionalized with (mannan-binding) concanavalin A (**d**–**l**). The graphs show the distribution of the Young modulus (**a**–**c**), the relationship between adhesion force and rupture distance (**d**–**f**), the distribution of the length of mannoprotein unfolded (**g**–**i**), and the frequency of adhesion events with varying adhesion forces (**j–l**). In panels **j–l, n.a**. stands for non-adhesion. Rows **A**, **B** and **C** show results for *Cyberlindnera jadinii*, *Blastobotrys adeninivorans* and *Wickerhamomyces anomalus*, respectively. The data were obtained from 3 cells (3072 curves were analyzed with JPK data processing software before fitting Gaussian curves on the distribution). The **blue circle** highlights the rupture distance at the adhesion force for ConA.

**Figure 6:**
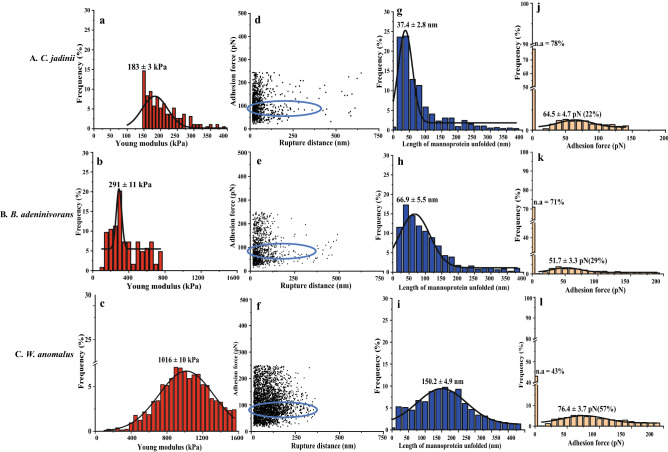
Probing cell wall architecture of three autolyzed yeast species (**A**–**C**) with Atomic Force Microscopy, using naked tips (**a**–**c**) or tips functionalized with (mannan-binding) concanavalin A (**d**–**l**). The graphs show the distribution of the Young modulus (**a**–**c**), the relationship between adhesion force and rupture distance (**d**–**f**), the distribution of the length of mannoprotein unfolded (**g**–**i**), and the frequency of adhesion events with varying adhesion forces (**j**–**l**). In panels **j–l, n.a**. stands for non-adhesion. Rows **A**, **B** and **C** show results for *Cyberlindnera jadinii*, *Blastobotrys adeninivorans* and *Wickerhamomyces anomalus*, respectively. The data were obtained from 3 cells (3072 curves were analyzed with JPK data processing software before fitting Gaussian curves on the distribution). The blue circle highlights the rupture distance at the adhesion force for ConA.

The elasticity of the yeast cells was determined by measuring the Young modulus using AFM with a silicon nitrite cantilever. *C. jadinii* exhibited the lowest Young modulus, 254 ± 12 kPa, while *B. adeninivorans* showed an intermediate value of 509 ± 7 kPa and *W. anomalus* showed the highest value (1126 ± 29 kPa) (Fig. 5a–c). The distributions were bimodal for ICJ and ABA, indicating that the cell elasticity is not homogenous among the cell population of these species. However, for all the three yeasts, autolysis reduced the Young modulus (Fig. 6a–c) and this effect was most pronounced for *B. adeninivorans*. This implies that cell permeability was modified during autolysis in the three yeast species.

The experiments with ConA-functionalized tips at the surface *vs* the force needed to break the interaction are presented in Fig. 5d–l and Fig. 6d–l. The adhesion frequency for inactivated yeast was 65%, 86% and 52%, for *C. jadinii*, *B. adeninivorans* and *W. anomalus,* respectively (Fig. 5j–l). Autolysis decreased the adhesion frequency for *C. jadinii* (22%) and *B. adeninivorans* (29%), but led to a minor increase in adhesion for autolyzed *W. anomalus* (57%) (Fig. 6j–l). The unbinding force or adhesion force of the interaction between the ConA-tip and the yeast cell surface was estimated to be in the range of 44–76 pN and there were no clear trends regarding the effect of autolysis on this force (Figs. 5j–l and 6j–l).

The length of mannoprotein unfolded (nm) differed between the yeast species and declined upon autolysis for *C. jadinii* and *B. adeninivorans*, but not for *W. anomalus*, which had the highest length of mannoprotein unfolded to begin with (Figs. 5g–i; 6g–i). The length of mannoprotein unfolded for inactivated *C. jadinii* and *B. adeninivorans* were around 70% and 20%, lower compared to inactivated *W. anomalus*, respectively. For autolyzed yeasts, the length of mannoprotein unfolded were 78% and 55% lower in *C. jadinii* and *B. adeninivorans*, compared to *W. anomalus*, respectively. Based on adhesion frequency and length of mannoprotein unfolded, it appeared that *B. adeninivorans* was most significantly affected by autolysis. The AFM experiments with ConA-functionalized tips also provided insight into the rupture distance, as shown in Figs. 5d–f and 6d–f. The rupture distance ranged from 0–400 nm for *C. jadinii* and *B. adeninivorans*, with a numerically wider distribution towards larger lengths for the autolyzed yeasts (Figs. 5d, e and 6d, e). In contrast, the rupture distance for *W. anomalus* ranged from 0–300 nm and became only slightly larger after autolysis (Figs. 5f and 6f).

Confocal micrographs (Figs. 3g–i; 4g–i), confirmed the expected specificity of ConA for mannans, as shown by the localized green coloration along the most exterior part of the yeast cell walls, where mannoproteins are predominately expected. Potential correlations between chemical composition of the cell wall and AFM-probed cell surface properties are presented in Supplementary Fig. S4. The data showed that there are significant positive correlations (*P* < *0.05*) between mannan content, cell wall thickness and Young modulus. Also, length of mannoprotein unfolded and adhesion force showed positive, but insignificant correlations (*P* > *0.05*) with the mannan contents of the yeasts. In contrast, there was a significant negative correlation between glucans and the AFM-derived parameters.

## Discussion

The current study shows that all the three yeasts could be used at 5% dietary inclusion level without compromising the performance of Atlantic salmon fry. Feed intake and biomass gain of fish were not affected by the dietary treatments; thus, we can assume that the observed differences in health-related parameters are mainly due to dietary treatments and not to differences in fish weight or feed consumption. The present study provides insight into the ability of the three yeast species to counteract SBMIE in Atlantic salmon fry, with particular focus on the effect of yeast autolysis.

Although dietary exposure to SBM is known to induce SBMIE in the seawater phase of the Atlantic salmon^4,9,15^, the effects are less severe during the freshwater phase^16,34,35^. In the current freshwater experiment, the histological findings were in accordance with previous studies performed in juveniles^16,34,35^, with only mild SBMIE symptoms detected. It has been suggested that the immaturity of intestinal functions may be responsible for the mild inflammatory changes observed in juveniles, compared to post-smolt salmons^34,35^. Despite mild symptoms, our results show that both AWA and ICU were efficient in alleviating SBMIE, as demonstrated by changes associated with widening of lamina propria, similar to those fed the FM control diet. ACJ and IWA had smaller non-significant effects on preventing SBMIE, while ICJ, IBA and ABA had no effects. These observations are in agreement with an earlier study of Grammes, et al.^15^, which showed that inactive dry *C. jadinii* and *Kluyveromyces marxianus* can be used to mitigate SBMIE in Atlantic salmon reared in seawater. Interestingly, the same yeast (ICU) used in the study of Grammes, et al.^15^, showed similar effect in our study, reinforcing our choice of a positive control. Our data also showed that the health beneficial effects of yeast depend on the type of yeast and the processing condition used after harvest.

Although the aetiology of SBMIE has been linked to the saponin content of SBM^4,6^, the exact mechanism of action is still debatable. Hitherto, amino acid and fatty acid metabolism, T-cell mediation, intestinal dysbiosis, and immune responses have been linked to SBMIE in Atlantic salmon^5,15,17,36,37^. Studies have shown a consistent abundancy of enteropathogenic bacteria^15,37^ and revealed that NOD-like receptors^15^ and Toll-like receptors^7^ are activated in fish suffering from SBMIE. Based on this information, we propose two possible pathways through which AWA, ICU, IWA and ACJ could alleviate SBMIE in the present experiment.

The first proposed mode of action is activation of the immune system by yeast cell wall components. In higher vertebrates, β-glucan exerts its mode of action by binding to dectin-1 receptors expressed on the surface of several innate immune cells such as dendritic cells, neutrophils, eosinophils, macrophages, monocytes and some T-cells^38^. It has been shown that the dectin-1 receptor synergizing with Toll-like receptors can modulate the production of TNFα in mice^39^. Similarly, the involvement of mannan in immune system activation has been reported in literature^40,41^ Mannan activates the immune system through C-type lectin receptors such as mannose receptors, dectin-2, dectin-3, galectin-3 and Toll-like receptors present in several immune cells^40,41^. In addition, a previous study has shown that loss of mannan in mutant yeasts reduces the levels of TNFα and IL-6 in human monocytes, which demonstrates the importance of mannan as an inducer of cytokine production in immune cells^42^. However, in fish, the presence of the dectin-1 receptor, along with the entire superfamily V of C-type lectin receptors, is still debatable^43^. Our results showed that only fish fed ICJ, ACJ, AWA and ICU had increased TNFα levels compared to SBM. The other yeast treatments did not promote increased production of TNFα compared to the SBM control group. TNFα is a pro-inflammatory cytokine, involved in an early stage of the immune response and has a key role during the inflammatory process by regulating the proliferation, migration and phagocytic activity of leukocytes^44^. Increased levels of TNFα may be related to the health beneficial effect of yeast in functional feeds, as reported in previous study^45^.

Increased TNFα production was counterbalanced by reduced Annexin 1 production in FM/ICJ/ICU fed fish, indicating that the immune responses were normalized in fish fed these diets. Our results showed that Annexin 1 production reduced in fish fed ICJ, ACJ, IBA, IWA, AWA and ICU diets, compared to SBM fed fish. Annexin 1 is an important marker for anti-inflammatory responses and has protective properties in the gut, as indicated previously being up-regulated in distal intestine of fish suffering from SBMIE^46^. Similarly, it has been reported that Annexin 1 was up-regulated during response to inflammatory bowel disease in humans^47^, which resemble SBMIE in fish, as reported in previous literature^15,48^ The positive correlation in immunological responses between the FM, ACJ, AWA and ICU diets suggested that the ability of ACJ, AWA and ICU to counteract SBMIE was linked to immune responses. Furthermore, the difference between ICJ-ACJ and IWA-AWA, in alleviating SBMIE, may be linked to accessibility of specific immune receptors (dectin-1, dectin-2 etc.) with yeast cell wall components^40,42^.

The second mechanism of action by which yeasts may counteract intestinal enteritis is through binding of its mannans with mannose-specific lectin-type receptors of enteropathogenic bacteria, thereby preventing adhesion of these bacteria to the surface glycoproteins of intestinal villi^49^. This is supported by our AFM experiment with ConA, which allow us to determine the adhesive (binding) capacity of mannan molecules on the surface of the yeast cells. The specificity of ConA for mannans was confirmed by the immunofluorescence analysis in this experiment, which is in contrast to the earlier approach where D-mannose was used to antagonize the surface of the AFM functionalized tip^50,51^. The binding capacity of yeast could be associated with the amount of mannan present on its cell wall. Our results showed that *W. anomalus* contained the highest amount of mannan, which may account for the improved protection against SBMIE, compared to the other yeasts.

The adhesion frequency influences the binding capacity of yeast cells and give an indication of distribution and accessibility of the mannoproteins on the surface of the yeast cell wall. High adhesion frequency suggests that mannoproteins are more accessible for interaction. In this study, adhesion frequency was reduced with autolysis in *C. jadinii* and *B. adeninivorans* yeast, but slightly increased in *W. anomalus*. This difference may explain the improved protection of AWA against SBMIE, compared with IWA. There is an indication that the length, type and flexibility of mannoprotein unfolded^52^ and the branching and structure of its α-mannoside residues^49,53^ may contribute to the adhesive properties of the yeast cell. In the present study, the length of mannoprotein unfolded ranged between 45–150 nm for the three yeasts and was higher than those observed for different strains of *S. cerevisiae*^52^. The difference in length of mannoprotein unfolded is an indication that the length of the mannan chains that made up the cell wall protein differed among the three yeasts, with IWA/AWA having the longest. Positive correlation between mannan content and length of mannoprotein unfolded indicates that mannan composition is linked to the stretching of mannoprotein on the surface of the yeast cells. Furthermore, the rupture distance gives useful information on the flexibility and extension of the mannoproteins^32,52^. The rupture distance ranged from 0–400 nm in *C. jadinii* and *B. adeninivorans*, with a slightly wider distribution towards larger lengths for the autolyzed yeasts. In contrast, rupture distance in *W. anomalus* ranged from 0–300 nm, and became slightly longer due to autolysis. The variation in rupture distance suggested that anchorage of mannoprotein differed among the yeasts^52^. This may indicate that the health beneficial effects of AWA were linked to its capability to adhere better with enteropathogenic bacteria, compared to the other yeasts.

Data from our AFM and cell wall thickness measurements indicated that *W. anomalus* had the highest mannoprotein levels, increased adhesion frequency when autolyzed and had the largest cell size compared to the other yeasts. Thus, the accessibility of mannoproteins can be a decisive factor for the protective effect of *W. anomalus* against SBMIE in Atlantic salmon fry. Moreover, the fact that the autolysis process increased this effect can be due to alteration of the cell wall surface, as shown by the change in the rupture distance. This observation support previous assertion of Firon, et al.^49^, who argued that the relationship between mannan concentration and pathogen adhesion is not always direct, indicating that involvement of other factors such as length, type and flexibility of mannoproteins, are key to the binding capacity of yeast. Although the present study indicates positive effects of AWA and ICU on SBMIE, further in vivo experiments, using the SBMIE model with Atlantic salmon in seawater is warranted to study the effect of different yeast strains and down-stream processing on gut health. Likewise, validation of these results with similar yeasts produced under industrial scale is recommended in the future. Furthermore, the diets in this trial were produced with cold-pelleting processing which differs from extrusion processing; however, in future studies, extruded diets are recommended to document the applicability of these yeasts in commercial salmon production.

In conclusion, this study demonstrates that the yeast strains *C. jadinii* and *W. anomalus* showed the most promising effect on gut health of Atlantic salmon, as demonstrated by histological changes based on widening of lamina propria, as well as changes in immune response parameters. Furthermore, processing by autolysis improved the health beneficial effect of the *W. anomalus*. Our data show that *C. jadinii* and *W. anomalus*, which has shown high productivity in previous fermentation studies, have potential for reducing SBMIE in Atlantic salmon. The results also showed that the amounts, length and accessibility of cell wall components (β-glucans and mannoproteins) could be decisive factors for the protective effects of yeast against SBMIE in Atlantic salmon fry. The functionality of yeast in counteracting intestinal enteritis in Atlantic salmon fry is dependent on the yeast species and the down-stream processing used during yeast production.

## Materials and methods

### Yeast production and processing

The yeasts *C. jadinii* and *B. adeninivorans* were cultivated in a company demonstration plant at 200 L scale (Biorefinery Demo, Borregaard AS, Sarpsborg, Norway), using a medium composed of enzymatic hydrolyzates of pre-treated spruce wood (*Picea abies*)^54^ and hydrolyzates of chicken by-products (Norilia, Oslo, Norway), as described in Lapeña, et al.^22^ *B. adeninivorans* was cultivated for 18.5 h in batch mode, while *C. jadinii* was cultivated for 42 h in fed-batch fermentation mode with the addition of wood sugars, urea, KH_2_PO_4,_ CaCl_2_·2H_2_O, MgSO_4_·7H_2_O and NaCl (See supplementary Fig. S1). *W. anomalus* yeast was cultivated in 20 L scale according to the protocols described in Lapeña, et al.^33^ For washing, the yeasts were separated by centrifugation and re-suspended in the same volume of 7 °C deionized water in a 30 L EINAR bioreactor system (Belach Bioteknik, Sweden), equipped with a helical impeller. The washed yeasts were then again centrifuged to obtain yeast creams with 12.5%, 5.5% and 15% dry matter contents for *C. jadinii* (CJ)*, B. adeninivorans* (BA) *and W. anomalus* (WA), respectively. Half of these microbial biomasses were dried and heat-inactivated by spray-drying using a SPX 150 MS (SPX Flow Technology, Denmark AS) spray-dryer with inlet and outlet temperatures of 180 °C and 80 °C, respectively. The spray-dryer was fitted with a co-current nozzle and the pump speed was set to auto and stabilized at around 35% of maximum speed of the pump. The other half of the yeast creams underwent autolysis by incubating the creams at 50 °C for 16 h in a 30 L EINAR bioreactor system, with constant stirring at 50 rpm using a helical impeller, followed by spray-drying using the same conditions as for the untreated yeast. Dried yeasts were kept at 4 °C until use.

### Formulation and production of fish feeds

Nine experimental diets were produced in this experiment. The diets were as follows: a fishmeal (FM) control; a diet with 40% SBM as a positive control; 6 treatment diets containing 40% SBM and 5% yeast ingredients [inactivated CJ (ICJ), autolyzed CJ (ACJ), inactivated BA (IBA), autolyzed BA (ABA), inactivated WA (IWA) and autolyzed WA (AWA)], respectively. An extra control diet containing 40% SBM and 5% of a reference preparation of *C. jadinii* (ICU) already described for its ability to counteract enteritis^15^ was also used in this trial. The feed formulation is as presented in supplementary Table S1. The diets were formulated to have a similar ratio of digestible protein to digestible energy, and to meet the nutrient requirements of Atlantic salmon as recommended by NRC^55^. To meet fish amino acid requirements, crystalline lysine and methionine were added to the diets due to the high inclusion of plant-based ingredients. All dry ingredients were mixed in a Spiry 25 dough mixer (Moretti Forni, Mondolfo, Italy). Gelatin was mixed in cold water and heated up to 60 °C in a microwave oven before mixing with dry ingredients and fish oil using the same mixer as above. The mash was cooled down to room temperature prior to cold-pelleting using a P35A pasta extruder (Italgi, Carasco, Italy). The pellets were dried (to about 91% dry matter content) in small experimental dryers at approximately 60 °C drying temperature and stored at 4 °C prior to feeding.

### Fish management and feeding

The fish experiment was conducted at the Fish Laboratory of Norwegian University of Life Sciences (NMBU, Ås, Norway), which is an experimental unit approved by the National Animal Research Authority, Norway (Permit No. 174). The experimental procedures were performed in accordance with the institutional and national guidelines under the applicable laws and regulations controlling experiments with live animals in Norway (regulated by the “Norwegian Animal Welfare Act” and “The Norwegian Regulation on Animal Experimentation” derived from the “Directive 2010/63/EU” on the protection of animals used for scientific purposes). The study was carried out in compliance with the ARRIVE guidelines.

In total, 1215 Atlantic salmon fry with an average start weight of 5.71 ± 0.05 g were sorted, batch weighed and randomly distributed into 27 fiberglass tanks (80 L) equipped with automatic feeders. Each tank was randomly stocked with 45 fish. Each diet was fed to triplicate tanks, 20% in excess based on feed consumption in each tank. Feeding was done twice a day with automatic feeders, and uneaten pellets were collected after each feeding from the outlet water settling on a screen for each tank. Daily feed intake was calculated from the dry weight of the feed given and the dry weight of recovered uneaten pellets, adjusted for feed recovery rate from fish tanks. Feeds were kept under refrigerated conditions (4 °C) throughout the experiment. Fish were exposed to a 24 h light regime and recirculated freshwater with an average temperature of 15.0 °C. The water flow was standardized to about 6 L min^−1^, and the oxygen content of the outlet water was kept within 8.2–10.1 mg L^−1^. The experiment lasted for 37 days, after which the fish were counted and grouped weighed to estimate the growth performance.

### Sampling procedure for fish tissue

For tissue sampling, six fish per tank were randomly selected, anesthetized with metacaine (MS-222; 50 mg L^−1^ water) and killed with a sharp blow to the head. The individual body weight of each fish was recorded and included in the total tank mean. Distal intestine and pyloric caeca tissues were collected from each fish and further processed, as described below. The distal intestine was opened longitudinally, the content was removed and the tissue was carefully divided into two parts: one part was fixed in 10% phosphate-buffered formalin for 24 h before storage in 70% ethanol until further processing for histological analysis; the second part was immediately snap-frozen in liquid nitrogen and stored at  − 80 °C for indirect enzyme-linked immunosorbent assays (ELISA). Pyloric caeca were treated in the same way as the distal intestine samples for histological analysis.

### Morphometric and histological examination of fish tissues

Formalin-fixed distal intestine and pyloric caeca samples were dehydrated in ethanol, equilibrated in xylene and embedded in paraffin using standard histological techniques. Longitudinal sections of approximately 6 µm in thickness were prepared. The sections were stained with hematoxylin, eosin and Alcian blue 8 GX. Changes in villi length were captured using a DMLS light microscope (Leica Microsystems, Wetzlar, Germany) equipped with a Leica E3 digital imaging camera and LAS EZ v4.9 software. Randomly selected villus of 18 distal intestine tissues from each dietary group (at least 80 measurements per group) was measured from the stratum compactum to the tip of the fold by ImageJ software. For histological evaluation, changes associated with intestinal tissues were blindly evaluated with a focus on the characteristic changes known for SBMIE in Atlantic salmon^9^. The histological scores were obtained through a semi-quantitative scoring system measuring changes in three morphological parameters: loss of supranuclear vacuoles in absorptive enterocytes; widening of lamina propria in mucosal folds; and increase of connective tissue between the base of folds and stratum compactum^9^. Each parameter was given a score of 1–5, where 1–2 represents normal morphology; 3–4 mild and moderate enteritis; and 5 for severe enteritis (Supplementary Fig. S2a, b). To measure changes associated with pyloric caeca*,* the longitudinal enterocytes area was selected (image 20×) and measured from the base to the apex. Measurement of enterocyte height was performed using the Easy Scan software. The total number and average mucous cell size in the caeca mucosal area were measured using the ImageJ software. The number of mucous cells was counted per 1 mm^2^ of the mucosal area (Supplementary Fig. S2c–e).

### Indirect ELISA of distal intestine tissues

Immunological parameters were analyzed using the distal intestine samples by indirect ELISA^56^. Briefly, distal intestine samples from nine fish per treatment were homogenized using metal beads and lysis buffer (Tris 20 mM, NaCl 100 mM, Triton X-100 0.05%, EDTA 5 mM, and protease inhibitor cocktail 1×, pH = 7.2). Subsequently, the homogenate was centrifuged at 12,000×g for 25 min at 4 °C. The supernatant containing soluble proteins was stored at  − 20 °C until use. The protein concentration was quantified using the BCA protein assay kit (Thermo Fisher Scientific) following the manufacturer’s instructions. Then, each sample was diluted in carbonate buffer (NaHCO_3_ 60 mM, pH 9.6) and seeded (in duplicate) in a 96-well plate (Maxisorp, Thermo Fisher Scientific) at 50 ng µL^−1^ (100 µL) for overnight incubation at 4 °C. After blocking with 5% Block solution (Bio-Rad) diluted in PBS, for 2 h at 37 °C, the plates were incubated for 90 min at 37 °C with the first antibody (Supplementary Table S2). Then, the second antibody-HRP (Thermo Fisher Scientific), at 1:7000 dilution, was added, followed by incubation for 1 h at 37 °C. Finally, the chromogenic substrate 3,3′,5,5′-tetramethylbenzidine (Invitrogen) was added (100 µL) followed by incubation for 30 min at room temperature. The reaction was stopped with 50 µL of 1 N sulfuric acid and absorbance at 450 nm was measured using a Spectramax microplate reader (Molecular Devices).

### Chemical analysis of yeast and fish feeds

The yeasts and diets were analyzed for dry matter by drying to constant weight at 105 °C (ISO 6496), for crude protein (N × 6.25) using CHNS Elemental Analyzer (Vario El Cube Elemental Analyzer system GmbH, Hanau, Germany), for crude lipid by Accelerated Solvent Extractor (ASE200, Dionex, California, USA) (ISO 6492) and for ash by incineration at 550 °C (ISO 5984). Gross energy content was determined using an adiabatic bomb calorimeter (Parr 1281; Parr Instruments, Moline, IL, United States), according to ISO (1998).

### Calculations for fish growth parameters

The average biomass gain, feed conversion ratio (FCR), and specific growth rate (SGR) were calculated according to the equations presented in Agboola, et al.^57^. Briefly, the biomass gain was calculated as the difference between the average final weight and the average initial body weight of fish per tank. The FCR was calculated as the ratio between average feed consumption per day and average biomass gain per day. The SGR was calculated as logarithm differences between average final and initial weight of fish divided by the experimental duration.

### Morphology and ultrastructure of the yeast cells

The ultrastructure of yeast cells was examined using a scanning electron microscope (SEM) and a transmission electron microscope (TEM). For each yeast, the SEM and TEM samples were taken before and after the autolysis process, i.e. before spray-drying. Three yeast samples per treatment were prepared according to the procedure described in Straume, et al.^58^ for SEM and TEM imaging. Samples for SEM were coated with Pt-Pl and examined in a Zeiss EVO 50 EP (Zeiss International, Germany) scanning electron microscope at an accelerating voltage of 15 kV in the secondary emission mode. The sections for TEM were examined in a FEI Morgagni 268 (FEI, USA) transmission electron microscope, and photographs were recorded with a VELETA camera. The imaging was performed at the Imaging Centre, Faculty of Biosciences, Norwegian University of Life Sciences (NMBU). The cell wall thickness was obtained by measuring the length of five random locations on the cell wall surface of twenty TEM micrographs of each yeast using ImageJ.

### Cell surface properties of yeast as determined by AFM

Atomic force microscopy (AFM) measurements were done following the protocol described in Schiavone, et al.^32^ and Schiavone, et al.^50^. Experiments were carried out with a Nanowizard III atomic force microscope (Bruker-JPK Instruments). The spring constants of each MLCT cantilever (Bruker) were determined using the thermal noise method^59^ and were found to be in the range of 10–20 pN nm^−1^. Yeast sample preparation was done by re-suspending the dry yeast mass in sodium acetate buffer (18 mM CH_3_COONa, pH 5.2, 1 mM CaCl_2_ and 1 mM MnCl_2_) and immobilized on polydimethylsiloxane (PDMS) stamps, as described in Dague, et al.^60^. 100 µL of yeast suspension was deposited on the PDMS stamps by convective/capillary assembly. Using bare AFM tips, AFM heights (expressed in nm) were recorded in Quantitative Imaging mode^61^ with a maximal force of 1 nN, at 20 °C in buffer solution. Elasticity of cells was determined from 3072 force curves recorded in force volume mode at an applied force to the surface of 0.5 nN and speed of approach and retraction of 2 µm s^−1^. Elasticity histograms were generated by analyzing the force-distance curves according to the Hertz model described in Schiavone, et al.^52^, with an indentation of 50 nm and considering a conical tip geometry with half-opening angle α of 0.31 rad and a Poisson ratio ν of 0.5.

To probe cell surface polysaccharides, AFM tips were functionalized with Concanavalin A (ConA) from *Canavalia ensiformis* (Sigma-Aldrich, L7647) via a silicon nitride dendritip as described in Jauvert, et al.^62^. To analyze the stretching of polysaccharides at the surface of the cell, elongation forces were stretched using the worm-like chain model introduced in Bustamante, et al.^63^, which describes the polymer as a curved filament. The contour length from this model represents the length of mannoprotein unfolded. At least three cells were analyzed for each treatment, representing a total of 3072 force curves for each treatment. The force curves were analyzed with the JPK data processing software (JPK BioAFM, Bruker Nano, Germany). All specific adhesion peaks were considered for the histograms, which were generated using the Origin 2020 software (OriginLab Northampton, MA, USA). A Gaussian distribution curve fitted on the histogram was used to determine the maximal values of Young modulus, length of mannoprotein unfolded and adhesion force for each yeast group.

### Quantification of yeast cell wall polysaccharides

The total polysaccharide content of the yeast cell wall was estimated without prior cell wall isolation according to the protocol described by François^64^. Briefly, the yeast samples were hydrolyzed with sulphuric acid and the released sugar monomers (mannose, *N*-acetylglucosamine and glucose) were quantified by high-performance anion-exchange chromatography with pulsed amperometric detection as described in Dallies, et al.^65^ and Hansen, et al.^23^. The content of β-glucan in the yeast samples was determined using a Megazyme kit (reference K-YBGL) and α-glucan was calculated as the difference between total glucan and β-glucan.

### Immunofluorescence analysis of yeast for determining mannan specificity for ConA

Approximately 200 mg of each spray-dried yeast was fixed with 10% formalin for 30 min at room temperature in Eppendorf tubes. Thereafter, the sample was centrifuged at 1000×g for 5 min at 4 °C and re-suspended in PBS. For fluorescence detection of mannan with ConA lectin, the sample was blocked for 1 h at room temperature with PBS containing 1% bovine serum albumin. Subsequently, the sample was incubated with 5 mg mL^−1^ of ConA-conjugated FITC (Sigma-Aldrich) for 1 h at room temperature in the dark. The samples were then gently layered on slides and allowed to dry for 10 min, before mounting in the Vectashield Medium (Vector Lab). Between all the steps of this procedure, the samples were washed in PBS. The slides were analyzed using a Zeiss LSM800 confocal microscope (Zeiss International, Germany).

### Statistical analysis

Fish performance, morphometric, histological and immune parameters were analyzed using the SPSS statistical software package version 26 (IBM Institute, Armonk, NY, USA). Fish performance, morphometric and immune response data were tested for treatment effects using one-way ANOVA. Significance difference (*P* < *0.05*) between means for fish performance and morphometric data were detected using the Tukey HSD test, whereas, for immune response parameters, Dunnett’s multiple comparison test was used for detecting significant differences. Data from morphometry measurements (villi length) was tested for normality by the Shapiro–Wilk test and homogeneity of variance using Levene’s test. Data from the histological evaluation were analyzed using a non-parametric Kruskal–Wallis test by ranks followed by Dunn’s multiple comparison test. Significance was set at *P* < *0.05*. The tank effect was considered for all parameters and found to have no influence on the statistical analyses. Correlation coefficients between the diets using five immune markers were examined using corrplot package in R. Likewise, correlations between dietary intake of yeast cell wall components and immune markers were determine using the same R package. Also, the correlations between cell wall components and AFM data were examined using the corrplot package in R (CRAN: http://cran.r-project.org/package=corrplot).

## Supplementary Information

Supplementary Information.

## Data Availability

The datasets generated during and/or analysed during this study are available upon reasonable request from the corresponding authors.
