# Author Correction: Impact of down-stream processing on functional properties of yeasts and the implications on gut health of Atlantic salmon (*Salmo salar*)

**DOI:** 10.1038/s41598-021-98424-8

**Published:** 2021-09-15

**Authors:** Jeleel Opeyemi Agboola, Marion Schiavone, Margareth Øverland, Byron Morales-Lange, Leidy Lagos, Magnus Øverlie Arntzen, David Lapeña, Vincent G. H. Eijsink, Svein Jarle Horn, Liv Torunn Mydland, Jean Marie François, Luis Mercado, Jon Øvrum Hansen

**Affiliations:** 1 Department of Animal and Aquacultural Sciences, Norwegian University of Life Sciences, P.O. Box 5003, 1432 Ås, Norway; 2 Lallemand SAS, 19 rue des Briquetiers, BP59, 31702 Blagnac, France; 3 Faculty of Chemistry, Biotechnology and Food Science, Norwegian University of Life Sciences, P.O. Box 5003, 1432 Ås, Norway; 4 TBI, Université de Toulouse, CNRS, INRAE, INSA, Toulouse, France; 5 Grupo de Marcadores Inmunológicos en Organismos Acuáticos, Pontificia Universidad Católica de Valparaíso, Avenida Universidad 330, Valparaíso, Chile; 6 LAAS-CNRS, Université de Toulouse, CNRS, Toulouse, France

Correction to: *Scientific Reports* https://doi.org/10.1038/s41598-021-83764-2, published online 24 February 2021

The original version of this Article contained errors in Figure 4, where panels (**SEM; b**) and (**SEM; c**) were a duplication of panel (**SEM; a**). The original Figure 4 and accompanying legend appear below.

**Figure 4:**
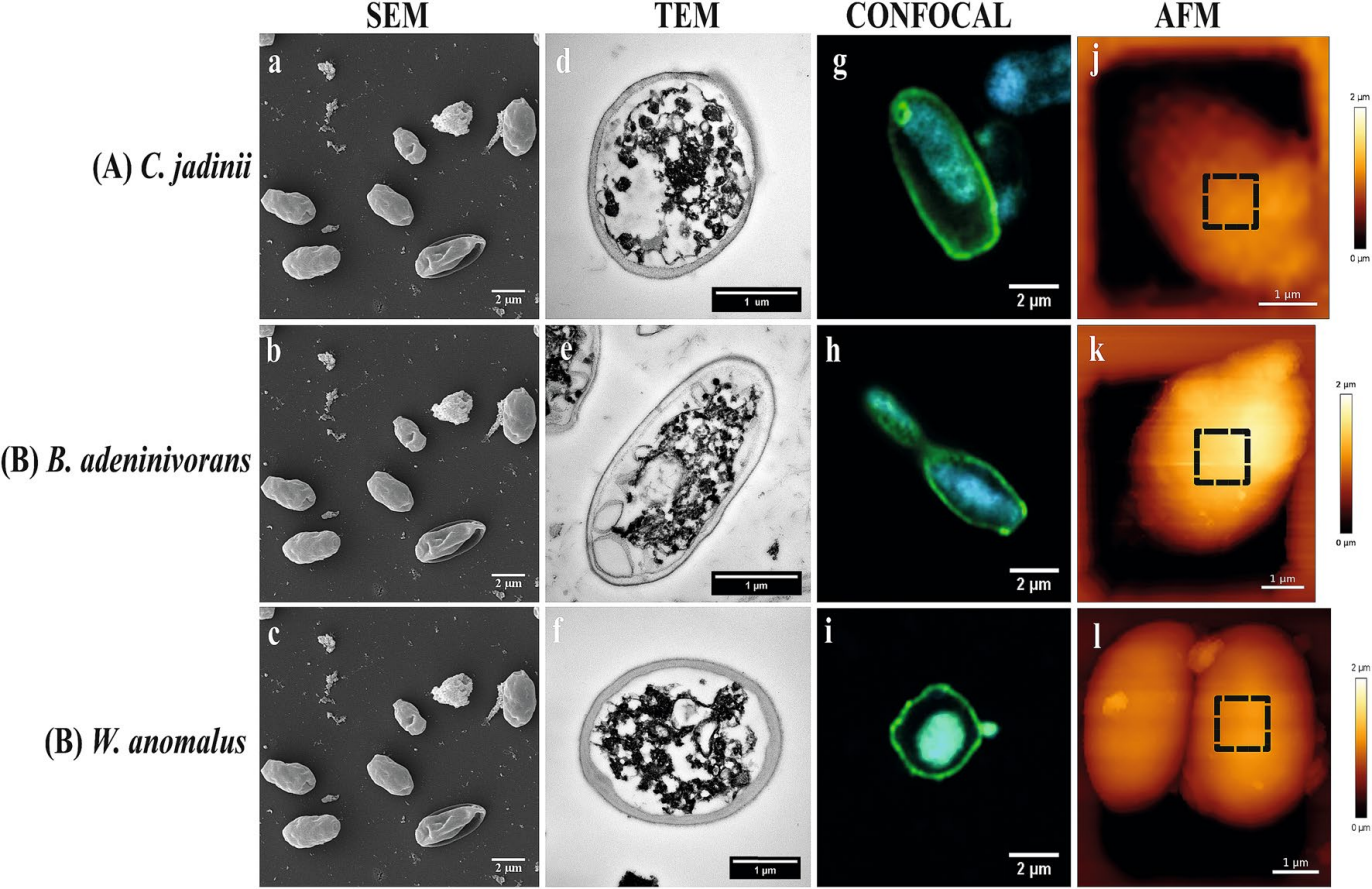
Cell surface architecture of three autolyzed yeast species (50 °C for 16 h) grown on sugars from lignocellulosic biomass. The pictures show Scanning Electron Microscopy (**SEM**; **a**–**c**), Transmission Electron Microscopy (**TEM**; **d**–**f**), Confocal microscopy (stained with concanavalin A-FITC for mannan) (**g**–**i**) and Atomic Force Microscopy (**AFM**; height) (**j**–**l**) micrographs of *Cyberlindnera jadinii* (**panel A**), *Blastobotrys adeninivorans* (**panel B**) and *Wickerhamomyces anomalus* (**panel C**). The SEM and TEM micrographs were taken on yeast creams (before drying), whereas the confocal and AFM micrographs were taken on dried yeast samples, as described in the ‘Material and Methods’. The dotted squares on the AFM height micrographs represent the spots where mapping was done for determination of the Young modulus and measurement of adhesion events, as described in ‘Material and Methods’.

The original Article has been corrected.

