# Supplementary material for: Impact of down-stream processing on functional properties of yeasts and the implications on gut health of Atlantic salmon (Salmo salar)
Source: Sci Rep. 2021 Feb 24;11:4496. doi: 10.1038/s41598-021-83764-2 (PMC7904851; doi:10.1038/s41598-021-83764-2)
Supplement: Supplementary file 1 — Supplementary Information. [file 41598_2021_83764_MOESM1_ESM.docx]

Impact of down-stream processing on functional properties of yeasts and the implications on gut health of Atlantic salmon (*Salmo salar*).

Jeleel Opeyemi Agboola^1*^, Marion Schiavone^2,4,6^, Margareth Øverland^1*^, Byron Morales-Lange^1^, Leidy Lagos^1^, Magnus Øverlie Arntzen^3^, David Lapeña^3^, Vincent G.H. Eijsink^3^, Svein Jarle Horn^3^, Liv Torunn Mydland^1^, Jean Marie François^4^, Luis Mercado^5^, and Jon Øvrum Hansen^1*^

^1^Department of Animal and Aquacultural Sciences, Norwegian University of Life Sciences, P.O. Box 5003, NO-1432 Ås, Norway.

^2^Lallemand SAS, 19 rue des Briquetiers, BP59, 31702 Blagnac, France.

^3^Faculty of Chemistry, Biotechnology and Food Science, Norwegian University of Life Sciences, P.O. Box 5003, NO-1432 Ås, Norway.

^4^TBI, Université de Toulouse, CNRS, INRAE, INSA, Toulouse, France.

^5^Grupo de Marcadores Inmunológicos en Organismos Acuáticos. Pontificia Universidad Católica de Valparaíso. Avenida Universidad 330, Valparaíso, Chile.

^6^ LAAS-CNRS, Université de Toulouse, CNRS, Toulouse, France.

**Supplementary Tables and Figures**

**Table S1**. Diet formulation and nutritional composition of the experimental diets. Diet formulation and composition are expressed in g/kg unless otherwise stated. The diets were fishmeal control, 40% soybean meal control, or 40% soybean meal + 5% yeast treatment diets*.

|  | FM | SBM | ICJ | ACJ | IBA | ABA | IWA | AWA | ICU |
| --- | --- | --- | --- | --- | --- | --- | --- | --- | --- |
| ***Diet formulation*** | | | | | |  |  |  |  |
| Fishmeal^a^ | 433.4 | 161.4 | 158.4 | 158.4 | 158.4 | 158.4 | 158.4 | 158.4 | 158.4 |
| Soybean meal^b^ | 0 | 400 | 400 | 400 | 400 | 400 | 400 | 400 | 400 |
| Wheat gluten meal^c^ | 170 | 136 | 111 | 111 | 111 | 111 | 111 | 111 | 111 |
| Yeast | - | - | 50 | 50 | 50 | 50 | 50 | 50 | 50 |
| Potato starch^d^ | 120 | 90 | 68 | 68 | 68 | 68 | 68 | 68 | 68 |
| Fish oil^e^ | 130 | 130 | 130 | 130 | 130 | 130 | 130 | 130 | 130 |
| Gelatin^f^ | 60 | 60 | 60 | 60 | 60 | 60 | 60 | 60 | 60 |
| Cellulose | 80 | - | - | - | - | - | - | - | - |
| MCP^g^ | 0 | 10.0 | 10.0 | 10.0 | 10.0 | 10.0 | 10.0 | 10.0 | 10.0 |
| Premix^h^ | 5.0 | 5.0 | 5.0 | 5.0 | 5.0 | 5.0 | 5.0 | 5.0 | 5.0 |
| L-lysine^i^ | - | 3.0 | 3.0 | 3.0 | 3.0 | 3.0 | 3.0 | 3.0 | 3.0 |
| DL-Methionine^j^ | - | 3.0 | 3.0 | 3.0 | 3.0 | 3.0 | 3.0 | 3.0 | 3.0 |
| Choline chloride^k^ | 1.5 | 1.5 | 1.5 | 1.5 | 1.5 | 1.5 | 1.5 | 1.5 | 1.5 |
| Yttrium oxide^l^ | 0.1 | 0.1 | 0.1 | 0.1 | 0.1 | 0.1 | 0.1 | 0.1 | 0.1 |
| ***Diet composition (analyzed)*** | | |  |  |  |  |  |  |  |
| Dry matter | 924.3 | 906.5 | 899.8 | 914.3 | 916.6 | 911.8 | 912.7 | 918.2 | 912.9 |
| Crude protein | 496.6 | 477.8 | 474.1 | 479.4 | 472.2 | 473.8 | 477.2 | 475.6 | 482.2 |
| Crude lipids | 191.5 | 166.5 | 162.5 | 171.0 | 169.0 | 164.0 | 163.5 | 166.5 | 167.5 |
| Ash | 71.5 | 60.0 | 63.5 | 64.9 | 63.2 | 62.8 | 61.8 | 61.9 | 62.1 |
| Gross Energy (MJ/kg) | 21.6 | 20.9 | 20.9 | 21.2 | 21.3 | 21.2 | 21.3 | 21.3 | 21.0 |
| DP:DE^m^ | 23.0 | 22.9 | 22.7 | 22.6 | 22.2 | 22.4 | 22.4 | 22.4 | 23.0 |

^a^LT fishmeal, Norsildmel, Egersund, Norway; ^b^Soybean meal, Denofa AS, Fredrikstad, Norway; ^c^Wheat gluten, Amilina AB, Panevezys, Lithuania; ^d^Lygel F 60, Lyckeby Culinar, Fjälkinge, Sweden; ^e^NorSalmOil, Norsildmel, Egersund, Norway; ^f^Rousselot 250 PS, Rousselot SAS, Courbevoie, France; ^g^Monocalcium phosphate, Bolifor MCP-F, Oslo, Norway Yara; ^h^Premix fish, Norsk Mineralnæring AS, Hønefoss, Norway. Per kg feed ; Retinol 3150.0 IU, Cholecalciferol 1890.0 IU, α-tocopherol SD 250 mg, Menadione 12.6 mg, Thiamin 18.9 mg, Riboflavin 31.5 mg, d-Ca-Pantothenate 37.8 mg, Niacin 94.5 mg, Biotin 0.315 mg, Cyanocobalamin 0.025 mg, Folic acid 6.3 mg, Pyridoxine 37.8 mg, Ascorbate monophosphate 157.5 g, Cu: CuSulfate 5H_2_O 6.3 mg, Zn: ZnSulfate 151.2 mg, Mn: Mn(II)Sulfate 18.9 mg, I: K-Iodide 3.78 mg, Ca 1.4 g; ^i^L-Lysine CJ Biotech CO., Shenyang, China; ^j^Rhodimet NP99, Adisseo ASA, Antony, France; ^k^Choline chloride, 70% Vegetable, Indukern SA., Spain; ^l^Y_2_O_3_. Metal Rare Earth Limited, Shenzhen, China.

^m^DP:DE = digestible protein: digestible energy ratio. Calculated using internal digestibility values of various ingredients.

*The diets are: **FM** – fishmeal-based; **SBM** – Soybean meal-based; 7 other diets containing 40% SBM and 5% of inactivated *Cyberlindnera jadinii* (**ICJ**), autolyzed *C. jadinii* (**ACJ**), inactivated *Blastobotrys adeninivorans* (**IBA**), autolyzed *B. adeninivorans* (**ABA**), inactivated *Wickerhamomyces anomalus* (**IWA**), autolyzed *W. anomalus* (**AWA**) and ref-*C. jadinii* (**ICU**).

**Table S2**. List of first antibodies used for indirect ELISA

| Marker | Source | Type | Dilution | Reference |
| --- | --- | --- | --- | --- |
| TNFα | Mouse | Polyclonal | 1:500 | Sahlmann, et al.^1^ |
| IFNγ | Mouse | Polyclonal | 1:500 | Sahlmann, et al.^1^ |
| CD83 | Rabbit | Polyclonal | 1:300 | Nombela, et al.^2^ |
| Annexin1 | Mouse | Polyclonal | 1:500 | Boltaña et al., accepted |
| IgM | Mouse | Monoclonal | 1:500 | Cat. No FM-190AZ-5, Ango |

**Table S3**. Daily consumption (mg dry matter per fish) of yeast cell wall components.^a^

| Diets^b^ | Mannans | Glucans | Chitin |
| --- | --- | --- | --- |
| FM | 0.00 | 0.00 | 0.00 |
| SBM | 0.00 | 0.00 | 0.00 |
| ICJ | 1.94 | 4.00 | 0.19 |
| ACJ | 1.99 | 3.00 | 0.33 |
| IBA | 1.90 | 2.43 | 0.41 |
| ABA | 1.49 | 1.79 | 0.36 |
| IWA | 3.17 | 2.03 | 0.36 |
| AWA | 3.01 | 1.78 | 0.48 |
| ICU | 1.68 | 2.84 | 0.34 |

^a^ Daily consumption of cell wall components was calculated from average daily feed intake (Supplementary Fig. S3b; dry matter) and the amount of each cell wall component in each yeast (Table 1; % dry mass).

^b^ The diets are: **FM** – fishmeal-based; **SBM** – Soybean meal-based and 7 other diets containing 40% SBM with 5% inclusion level of inactivated *Cyberlindnera jadinii* (**ICJ**), autolyzed *C. jadinii* (**ACJ**), inactivated *Blastobotrys adeninivorans* (**IBA**), autolyzed *B. adeninivorans* (**ABA**), inactivated *Wickerhamomyces anomalus* (**IWA**), autolyzed *W. anomalus* (**AWA**) and ref-*C. jadinii* (**ICU**).


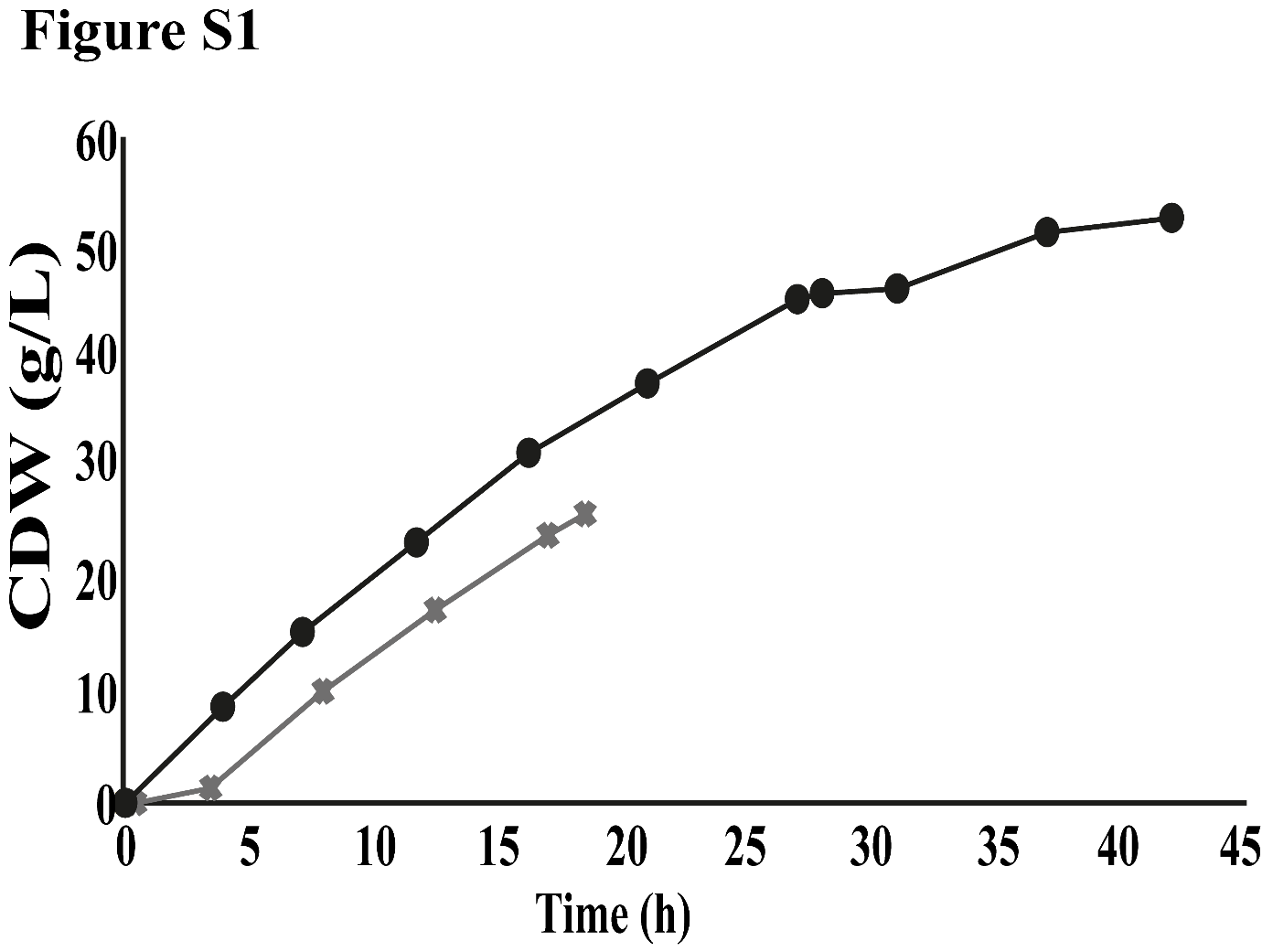


**Figure S1.** Cell dry weight concentrations (CDW) in batch cultivation (200 L working volume) of *B. adeninivorans* (**grey curve**) and fed-batch cultivation of *C. jadinii* strain (**black curve**). For fed batch cultivation starting volume was 100 L with addition of more glucose at 7.2, 11.8, 16.3 and 21 h (5 kg glucose each time) yielding a final working volume of 193 L. Initial glucose concentration in both fermentations was 50 g/L. Temperature and pH of the fermentations were 30 ⁰C and 5.0, respectively. Aeration and stirrer speed were constant at 200 L/min and 300 rpm, respectively. Final cell yields (Y_X/glc_) for *B. adeninivorans* and *C. jadinii* were 0.52 and 0.41 g dry cells per g of glucose, respectively.


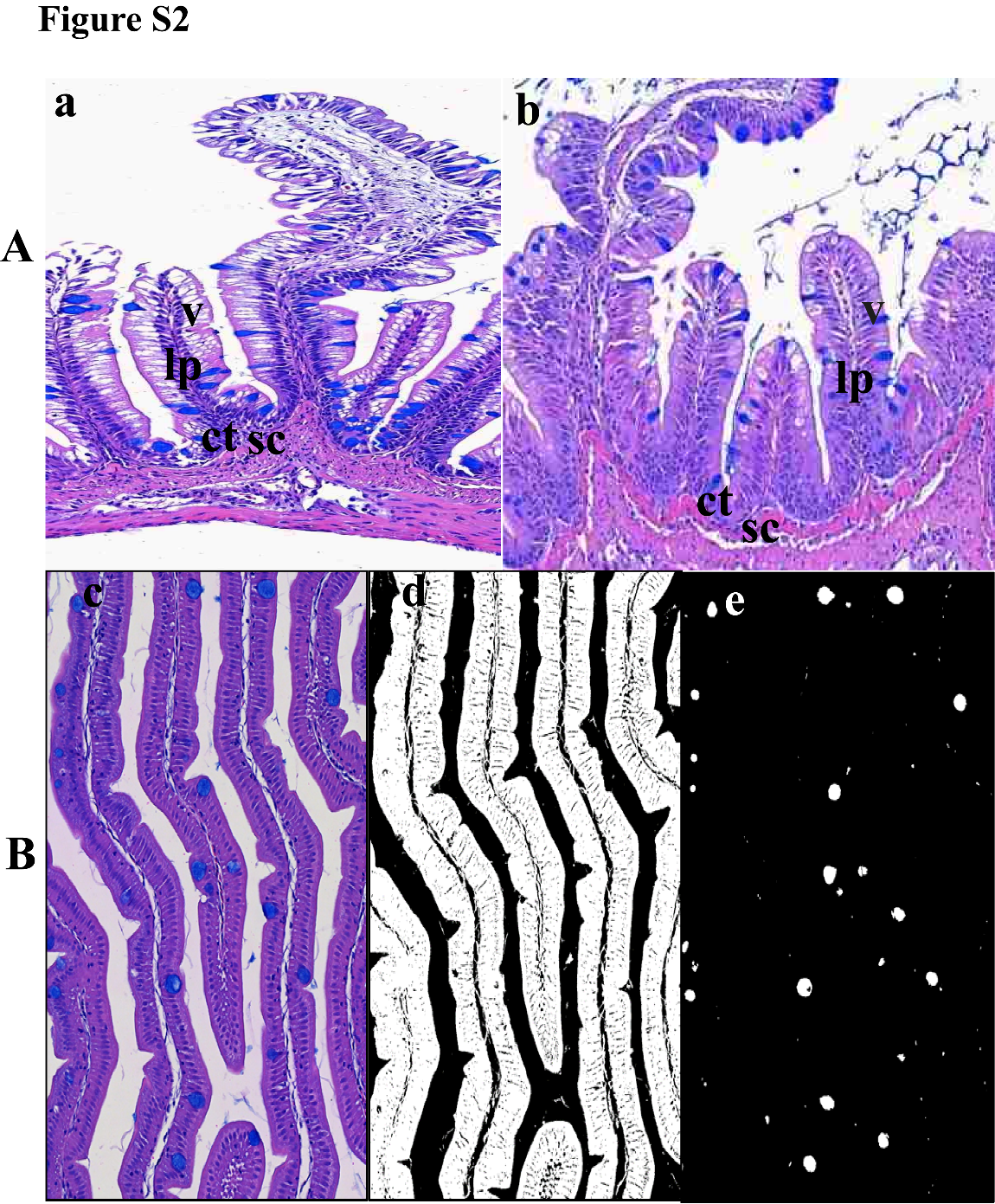


**Figure S2.** Example of images used for measuring the histopathological changes in the distal intestine (**A**) and pyloric caeca (**B**) of Atlantic salmon fry. The changes in the distal intestine were measured by assigning a score between “**1-5**” based on observed changes in loss of vacuoles (**v**) in absorptive enterocytes, widening of lamina propria (**lp**) in mucosal folds and increase of connective tissue (**ct**) between base of folds and stratum compactum (**sc**). A score of “**1-2**” represents normal morphology; “**3-4**” mild and moderate enteritis; whereas “**5**” denotes severe enteritis. Normal (**a;** score = 1) and distal intestine with mild/moderate (**b;** score =3) enteritis are shown. For the pyloric caeca (**B**), an example of interface flow for the quantification of mucous cells in mucosal area is presented. The image (**c)** represents the original image of the pyloric caeca, (**d)** is a selection mask, and (**e)** is used for cell quantification.


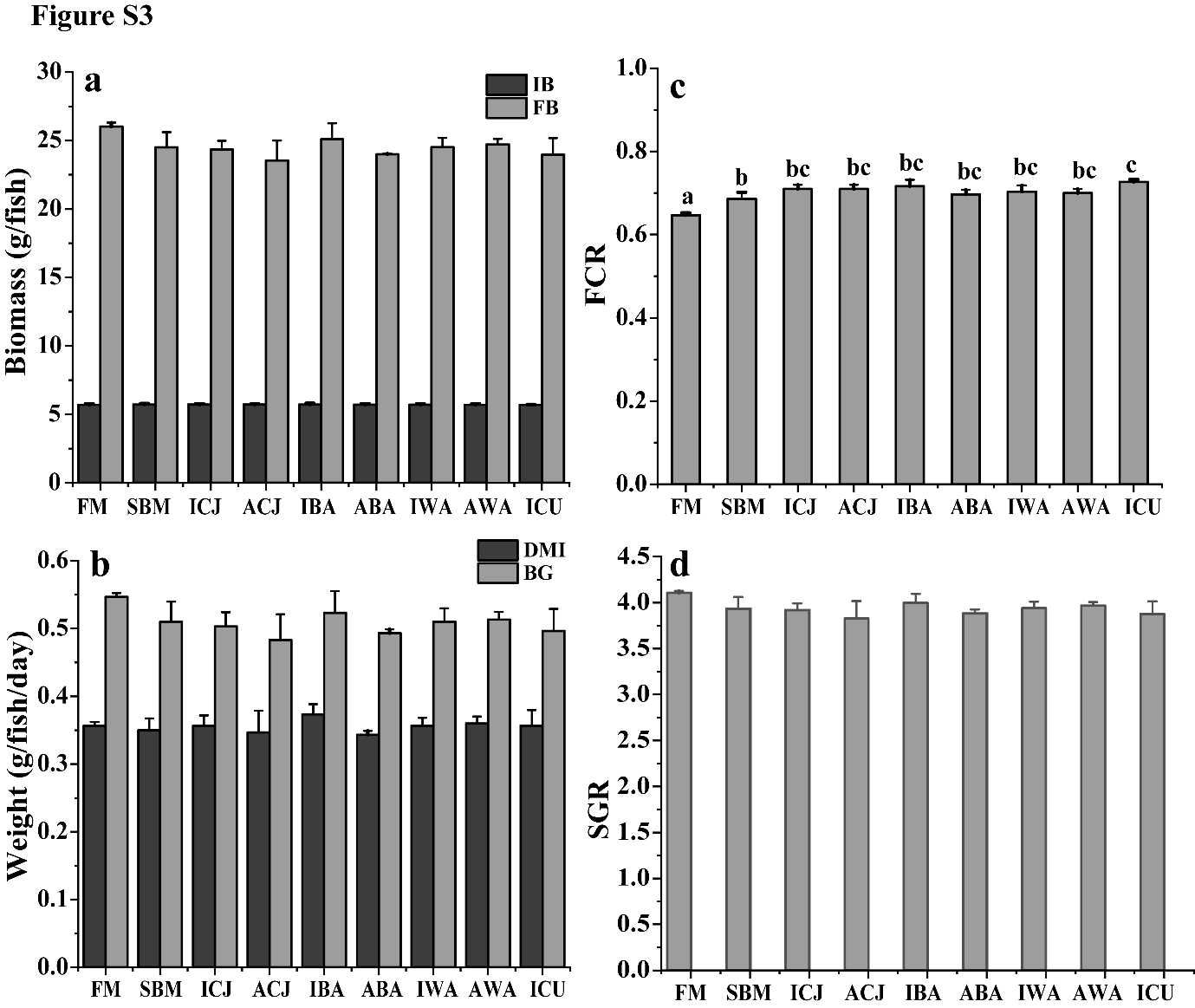


**Figure S3.** Growth performance of Atlantic salmon fry fed with soybean meal-based diets with yeasts. The growth indices are represented by; **a** – initial (**IB**) and final (**FB**) biomass; **b** – dry matter intake (**DMI**) and biomass gain (**BG**); **c** – feed conversion ratio (**FCR**); and **d** – specific growth rate (**SGR**). The experiment diets are: **FM** – fishmeal-based; **SBM** – Soybean meal-based and 7 other diets containing 40%SBM with 5% inclusion level of inactivated *Cyberlindnera jadinii* (**ICJ**), autolyzed *C. jadinii* (**ACJ**), inactivated *Blastobotrys adeninivorans* (**IBA**), autolyzed *B. adeninivorans* (**ABA**), inactivated *Wickerhamomyces anomalus* (**IWA**), autolyzed *W. anomalus* (**AWA**) and ref-*C. jadinii* (**ICU**). The letters **a**, **b** and **c** directly above the bar charts denote treatment(s) with a statistical difference (*P < 0.05).*


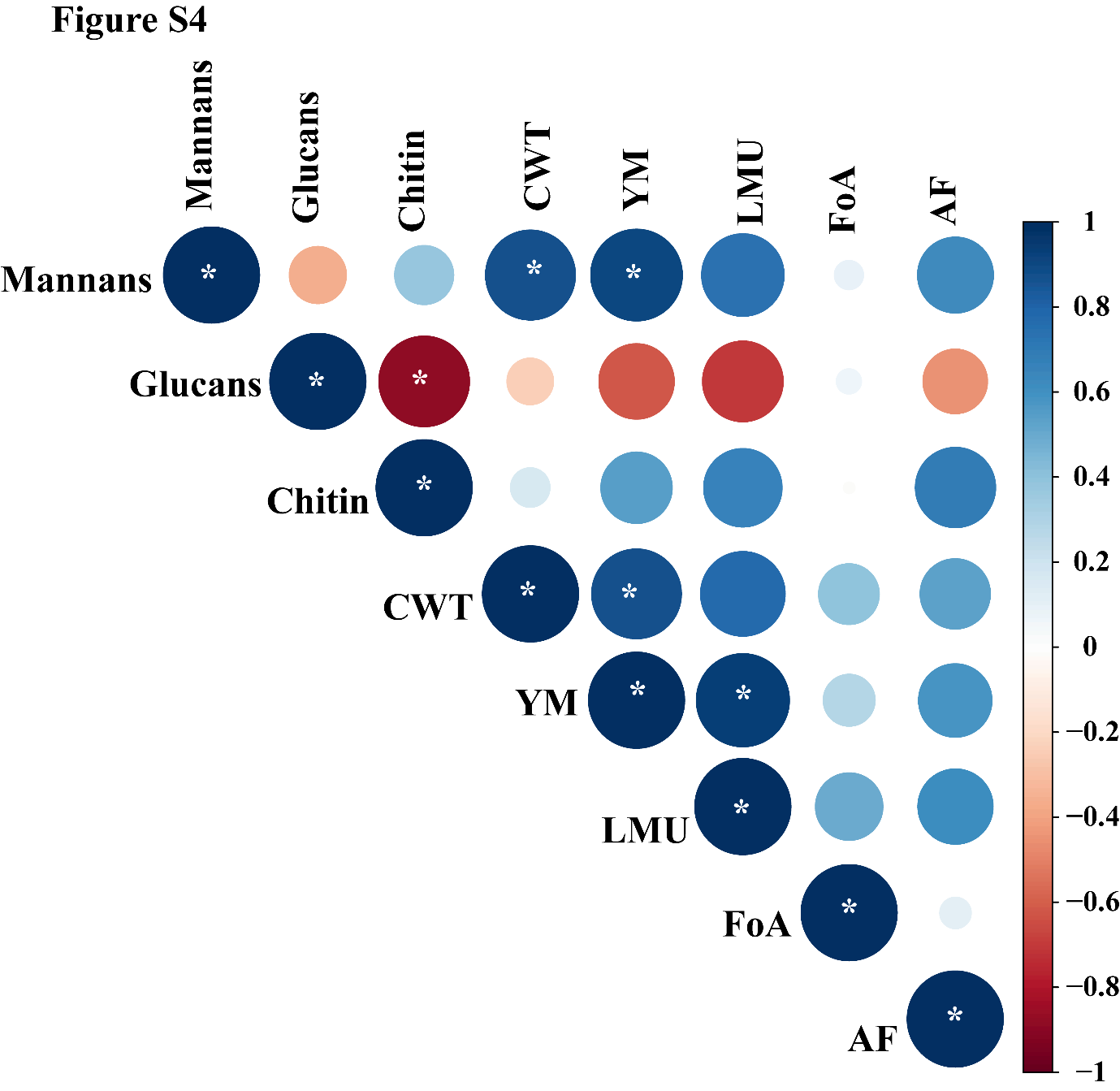


**Figure S4**. Correlation matrix showing relationships between the cell wall components (glucans, mannans and chitin) of yeasts and cell wall thickness (**CWT**); Young modulus (**YM**); length of mannoprotein unfolded (**LMU**), frequency of adhesion (**FoA**) and adhesion force (**AF**) obtained from atomic force microscopy. **Positive** correlations are displayed in **blue** and **negative** correlations in **red** color. Both **color intensity** and the **size of the circle** are proportional to the correlation coefficients. Correlations with significant values at P < 0.05 are shown with *****.

**REFERENCES**

1. Sahlmann, C. *et al.* Yeast as a protein source during smoltification of Atlantic salmon (*Salmo salar* L.), enhances performance and modulates health. *Aquaculture* **513**, 1-10 (2019).

2. Nombela, I. *et al.* Rainbow trout red blood cells exposed to viral hemorrhagic septicemia virus up-regulate antigen-processing mechanisms and MHC I&II, CD86, and CD83 antigen-presenting cell markers. *Cells* **8**, 1-29 (2019).
